# Characterization and Dimethyl Phthalate Flocculation Performance of the Cationic Polyacrylamide Flocculant P(AM-DMDAAC) Produced by Microwave-Assisted Synthesis

**DOI:** 10.3390/molecules25030624

**Published:** 2020-01-31

**Authors:** Peng Zhang, Dong Qin Zhao

**Affiliations:** 1 College of Environmental Science and Engineering, Taiyuan University of Technology, Taiyuan 030024, China; 2 College of Civil Engineering, Hunan University of Science and Technology, Xiangtan 411201, China; 18302316621@163.com

**Keywords:** acrylamide, intrinsic viscosity, flocculant, dimethyl phthalate

## Abstract

A composite flocculant P(AM–DMDAAC) was synthesized by the copolymerization of acrylamide (AM) and dimethyl diallyl ammonium chloride (DMDAAC). By using microwave (MV) assistance with ammonium persulfate as initiator, the synthesis had a short reaction time and yielded a product with good solubility. Fourier-transform infrared spectroscopy, scanning electron microscopy, and differential thermal analysis–thermogravimetric analysis were employed to determine the structure and morphology of P(AM–DMDAAC). The parameters affecting the intrinsic viscosity of P(AM–DMDAAC), such as MV time, mass ratio of DMDAAC to AM, bath time, reaction temperature, pH value, and the dosages of ammonium persulfate initiator, EDTA, sodium benzoate, and urea were examined. Results showed that the optimum synthesis conditions were MV time of 1.5 min, m(DMDAAC):m(AM) of 4:16, 0.5 wt‰ initiator, 0.4 wt‰ EDTA, 0.3 wt‰ sodium benzoate, 2 wt‰ urea, 4 h bath time, reaction temperature of 40 °C, and pH of 2. The optimal dimethyl phthalate (DMP) removal rate can reach 96.9% by using P(AM–DMDAAC), and the P(AM–DMDAAC) had better flocculation than PAM, PAC, and PFS.

## 1. Introduction

Dimethyl phthalate (DMP), a commonly used plastic plasticizer, is widely used in polyethylene plastic, artificial leather, hose, daily necessities, and medical supplies [1]. DMP is an environmental hormone, an important global organic pollutant, and widely used as a raw material for the production of pesticide carriers, dyes, cosmetics, lubricants and defoamers [2,3,4]. When the concentration of DMP exceeds the limit value, aquatic animals and human health are affected [5,6]. With the increased use of plastics and other products, the pollution area and concentration of DMP increase annually. If necessary technical measures are not employed in time to reduce the concentration of DMP in water, the safety of drinking water may be affected [7]. Water can be treated via several physicochemical methods, such as adsorption, solvent extraction, wet oxidation, heterogeneous photocatalysis, biological treatments, and advanced oxidation [8,9,10,11,12]. However, routinely available methods suffer from serious drawbacks, including high cost and the formation of hazardous byproducts requiring management and disposal. As an alternative, flocculation is an environmentally friendly, cost-effective, and an increasingly important process in pollution control [13,14,15,16]. Flocculation is a usual step in water treatment and the flocculant is the core of flocculation technology, so it is very important to choose a suitable flocculant [17,18,19,20,21,22]. Consequently, priorities for flocculant and coagulation performance include high efficiency, low cost, and low environmental impact [23,24,25,26].

Currently, many organic flocculants are available, including synthetic and natural flocculants, such as cationic polyacrylamides (CPAM), anionic polyacrylamides, chitosan and its derivatives, and starch and its derivatives [27,28,29,30]. Specifically, CPAMs are the subject of increased attention because of their high efficiency in the purification of drinking water and wastewater. The CPAM family of flocculants are based on acrylamide (AM), which is a cost-effective reactive monomer in free radical polymerization with extremely good water solubility. As a derivative of polyacrylamide (PAM), the unique side chain structure and high-density charge of AM are suitable for the treatment of wastewater containing fine particles, suspended solids, organics, and colloids [31,32,33]. These typical organic flocculants have electropositive properties consistent with the presence of acryloyl oxygen ethyl trimethyl ammonium chloride (DAC), methyl acryloyl oxygen ethyl trimethyl ammonium chloride (DMC), and dimethyl diallyl ammonium chloride (DMDAAC) functional groups. The conventional methods for the synthesis of CPAM use a free radical initiator to generate free radical sites on the backbone polymer [34,35,36,37,38]. The reaction of aqueous polymerization is initiated by several methods, including direct heat, X-rays, microwave (MV), and ultraviolet radiation. In comparison with other methods, MV initiation has many advantages, such as short reaction time, low dosage of initiator, and low reaction temperature. MV initiation involves MV-initiated (no chemical initiator) and MV-assisted (low dosage of initiator) syntheses.

This study aimed to investigate the possibility of synthesizing the P(AM–DMDAAC) flocculant by MV-assisted initiated polymerization with AM and DMDAAC as monomers. The synthesis conditions, namely, MV time (0~4 min), mass ratio of DMDAAC to AM (2:18~4:16), water bath time (2~6 min), reaction temperature (30~70 °C), pH value (2~6), and the doses of ammonium persulfate (0.2~0.6‰), EDTA (0.3~0.7‰), sodium benzoate (0.2~0.6‰), and urea (0.1~0.5‰) were investigated to obtain the optimal polymer with high intrinsic viscosity (η). Fourier transform infrared (FTIR) spectroscopy, scanning electron microscopy (SEM), and differential thermal analysis-thermo gravimetric analysis (DTA-TGA) were used to study the structural characteristics. Finally, the flocculation performance of P(AM–DMDAAC) was evaluated on the basis of its DMP removal efficiency in a synthetic water sample.

## 2. Results and Discussion

### 2.1. Synthesis of P(AM–DMDAAC)

The intrinsic viscosity (*η)*, a major determinant in controlling P(AM–DMDAAC) performance [39], was used to assess the effectiveness of P(AM–DMDAAC) prepared in this study. The parameters that affect *η*, such as MV time, mass ratio, bath time, temperature, pH, and doses of ammonium persulfate, ethylenediaminetetraacetic acid (EDTA), sodium benzoate, and urea, were examined.

#### 2.1.1. Effect of MV Time on *η*

**Figure 1:**
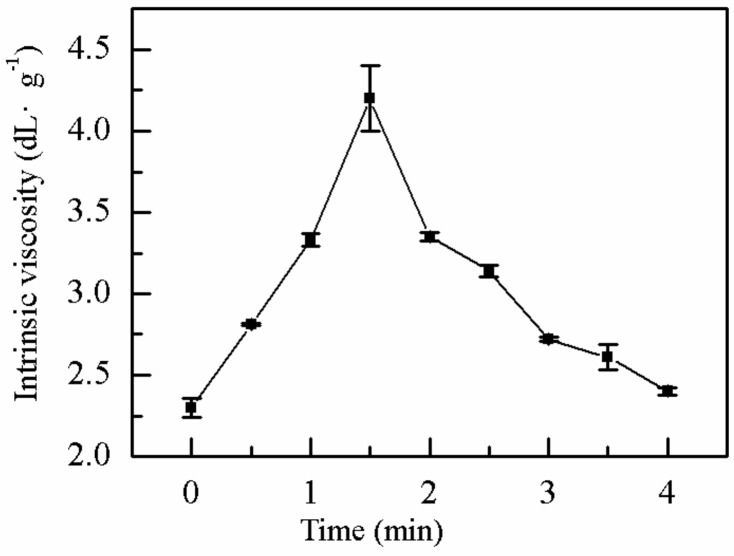
Effect of microwave time on *η.*

MV power is one of the most important factors that affect the properties of polymer synthesis. Excessive MV power may cause a fast polymerization. The effect of microwaves on reactant ions and polar molecules with different electrical properties is to make them move in the opposite direction, so that the relative motion energy and collision frequency of reactant molecules are increased, leading to the increase of pre exponential factor of microwave reaction system, and the effective collision of reaction is also increased in most cases [40,41], with an abrupt increase in the temperature of the system, and inefficient dissipation of the heat of polymerization. MV power was fixed at 20 W to minimize these effects. The effect of MV time on the *η* of the polymer was investigated. As shown in Figure 1, the effect of MV time on the *η* of the polymer was investigated at m(DMDAAC):m(AM) = 4:16, ammonium persulfate dose of 0.5‰ (*w*/*v*), EDTA dose of 0.4 ‰ (*w*/*v*), sodium benzoate proportion of 0.3 ‰ (*w*/*v*), urea dose of 0.2 ‰ (*w*/*v*), bath time of 4 h, bath temperature of 40 °C, and pH value of 2. The *η* of the polymer exhibited a peak at 1.5 min and reached a maximum of 4.20 dL·g^−1^. At low MV time, sufficient temperature and the MV effect in the reaction system were hardly obtained, indicating that the polymerization chain reaction was not established. At prolonged MV time, the reaction rate increased until reaching the optimum value and then degraded due to the collision probability between free radicals. At the same time, the chain reaction was easily terminated, thus decreasing the *η* of the P(AM–DMDAAC). Therefore, the optimum MV time was identified as 1.5 min.

#### 2.1.2. Effects of the Monomer Mass Ratio on *η*

**Figure 2:**
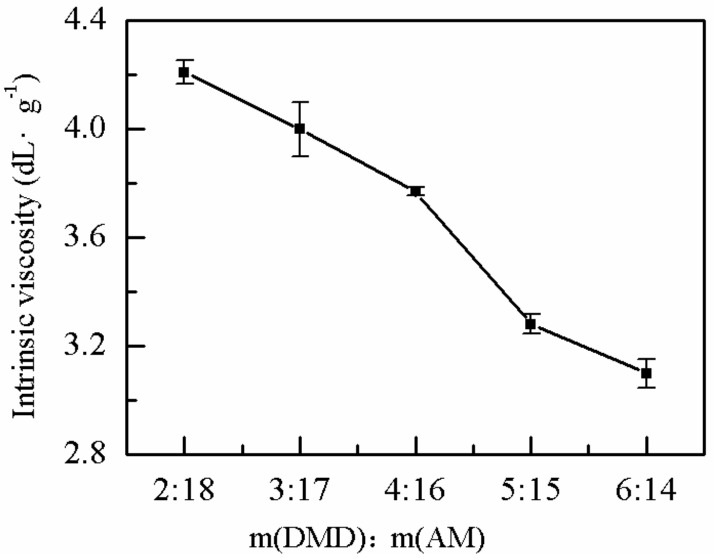
Effect of monomer mass ratio on η.

The effects of the mass ratio of DMDAAC to AM on the *η* of P(AM–DMDAAC) was explored. The effect of monomer mass ratio on *η* was evaluated at MV time, ammonium persulfate dose, EDTA dose, sodium benzoate proportion, urea dose, bath time, bath temperature, and pH value of 1.5 min, 0.5‰ (*w*/*v*), 0.4 ‰ (*w*/*v*), 0.3 ‰ (*w*/*v*), 0.2 ‰ (*w*/*v*), 4 h, 40 °C, and 2, respectively. As shown in Figure 2, the *η* of the polymer decreased with increasing proportion of DMDAAC monomer. When m(DMDAAC):m(AM) was 2:18, the *η* of the polymer reached the maximum (4.21 dL·g^−1^) because the chemical reactivity of the DMDAAC cationic monomer was lower than that of AM. When the initiator used was ammonium persulfate, the chemical reactivity ratios of the DMDAAC monomer to the AM monomer were 0.58 and 6.7, respectively, indicating that the activity of the AM monomer was higher than that of the DMDAAC monomer. A higher proportion of DMDAAC monomer resulted in a longer time required to reach the polymerization temperature, leading to incomplete monomer polymerization. Hence, the *η* of the polymer decreased with increasing proportion of the DMDAAC monomer. The cationic degree was also an important factor that influences the choice of polymer. Generally, a higher degree of cationic monomer resulted in better water treatment effect [42]. Therefore, the optimum m(DMDAAC):m(AM) was 4:16.

#### 2.1.3. Effect of Ammonium Persulfate Dose on *η*

**Figure 3:**
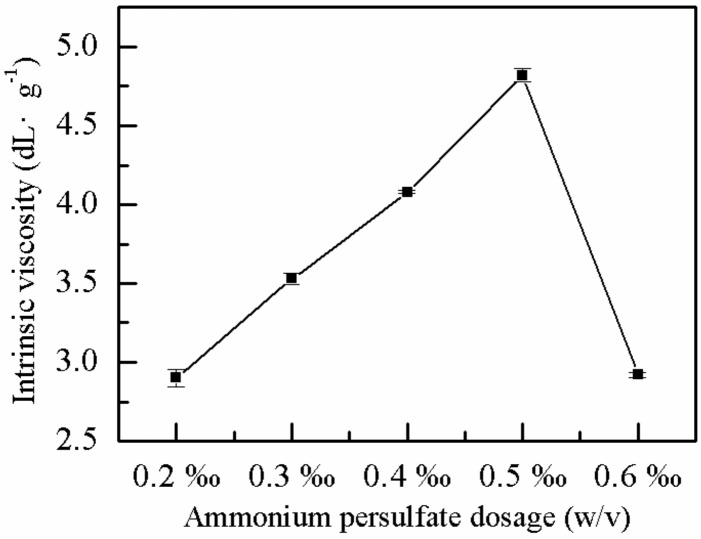
Effect of ammonium persulfate dose on *η*.

The effects of the dosage of ammonium persulfate on the *η* of P(AM–DMDAAC) was explored, and the other factors were constant. As shown in Figure 3, the *η* of the polymer exhibited an optimal point with increasing proportion of ammonium persulfate. The *η* of the polymer was highest (4.80 dL·g^−1^) when the proportion of the ammonium persulfate initiator was 0.5‰ (*w*/*v*). When the proportion of the initiator was low, the concentration of free radicals and the reaction point were low. Hence, the chain reaction was difficult to initiate and sustain, and the *η* of polymer was low. Increasing the proportion of the initiator increased the concentration-free radicals and the number of the reaction centers, which increased the reaction rate and the *η* of the polymer. However, when the initiator proportion was very high, the excessive number of free radicals increased the probability of collision and chain termination [43]. Moreover, a fast reaction rate may lead to a sudden rise in temperature with a high susceptibility of implosion and low *η* of the polymer. Therefore, the optimum initiator ammonium persulfate dose was 0.5‰ (*w*/*v*).

#### 2.1.4. Effect of EDTA Dose on *η*

**Figure 4:**
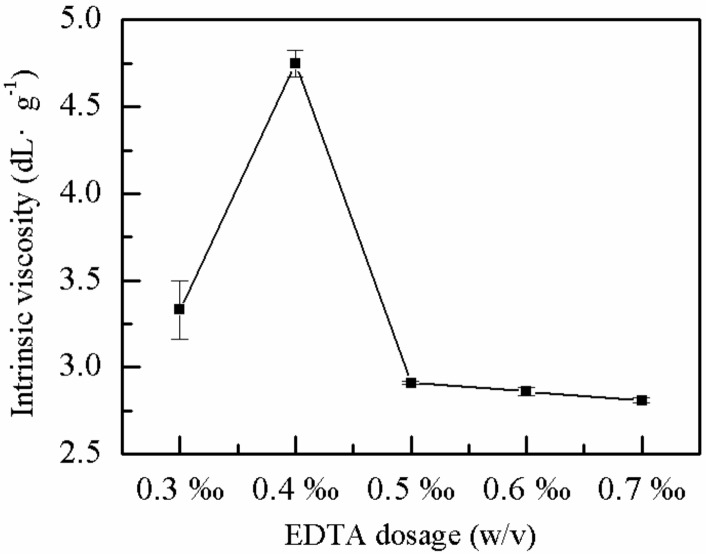
Effect of EDTA dose on *η*.

The industrial-grade AM monomer contains metal ions, such as Cu^2+^ and Fe^2+^, which can consume primary radicals during reaction. These metal ions may hinder chain propagation and increase the induction period. The presence of EDTA in a complex with Cu^2+^ and Fe^2+^ may eliminate the influence of metals on the reaction and increase the *η* of the polymer. The effects of the proportion of EDTA on the *η* of P(AM–DMDAAC) were explored with all other conditions given. As shown in Figure 4, the *η* of the polymer showed a similar optimum with the increasing proportion of the EDTA. The *η* of the polymer was highest (4.7 dL·g^−1^) when the proportion of EDTA was 0.4 ‰ (*w*/*v*). At low EDTA dosage, the metal ions in the reaction system, such as Cu^2+^ and Fe^2+^, were in excess, which hindered the polymerization reaction. Conversely, when the proportion of EDTA was high, the probability of chain transfer reaction increased, which did not enhance the *η*. The optimum dose of EDTA was 0.4 ‰ (*w*/*v*).

#### 2.1.5. Effect of Sodium Benzoate on η

**Figure 5:**
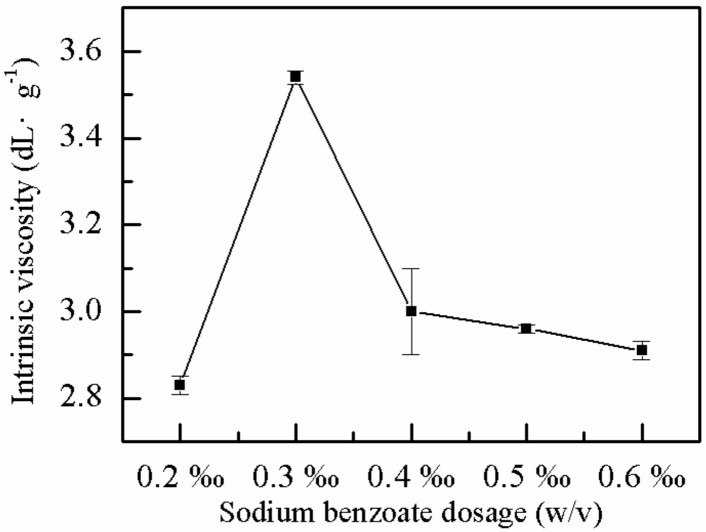
Effect of sodium benzoate on *η*.

Sodium benzoate is a chain transfer agent, which can prevent the crosslinking of the polymer effectively and ensure the growth of the chain length. The effects of sodium benzoate on the *η* of P(AM–DMDAAC) were explored with all other conditions given. As shown in Figure 5, the optimal *η* of the polymer (3.54 dL·g^−1^) was observed when the proportion of the sodium benzoate was 0.3 ‰ (*w*/*v*). When the proportion of sodium benzoate was low and insufficient to consume excess free radicals or to prevent the reaction system from being crosslinked, the *η* of the polymer decreased. Conversely, when the proportion of sodium benzoate was high, which can lead to the complete consumption of parts of the free radicals in the reaction system, the chain growth in the polymerization reaction was hindered, and the *η* of the polymer decreased [44].

#### 2.1.6. Effect of Urea Dose on η

**Figure 6:**
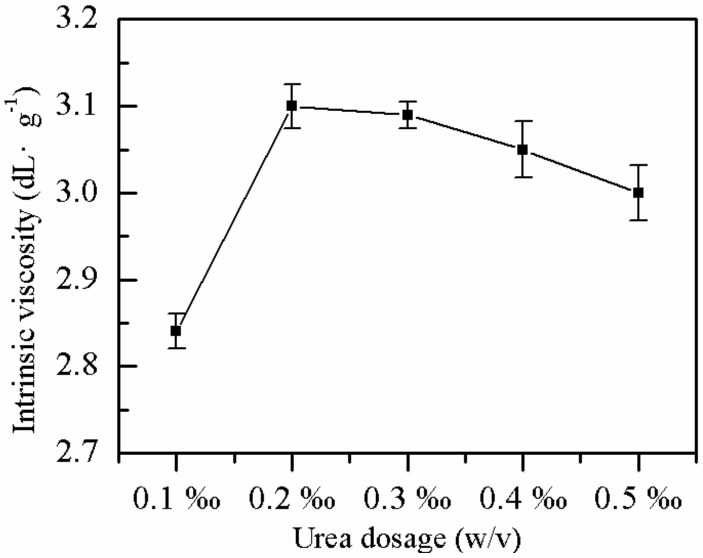
Effect of urea dose on *η*.

As shown in Figure 6, the effects of the consumption of urea on the *η* and solubility of P(AM–DMDAAC) showed optimal conditions with increasing urea while keeping the remaining factors constant. When 0.2 ‰ (*w*/*v*) urea was added, the *η* of the polymer was highest (3.1 dL·g^−1^) as the low dose of urea assisted the reducing agent, which involved the redox reaction and favored chain growth. Thus, the *η* of the polymer increased. When the urea dose was high, chain transfer occurred, which hindered chain growth and reduced the *η* of the polymer. When the amount of urea increased from 0.1 ‰ to 0.5 ‰, the dissolution time decreased from 149 min to 99 min.

#### 2.1.7. Effect of Bath Time on η

**Figure 7:**
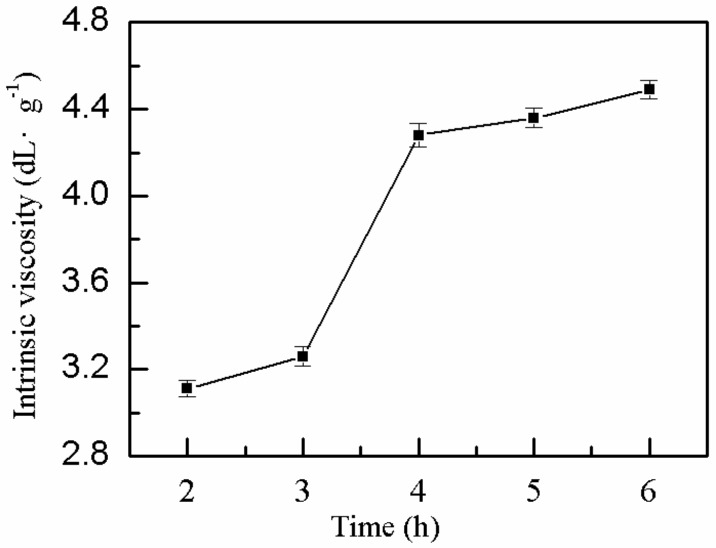
Effect of bath time on *η*.

The effect of bath time on the *η* of P(AM–DMDAAC) was explored, whereas the remaining factors were kept constant. The *η* of the polymer increased with increasing water bath time, but the growth reached a plateau after extension (Figure 7). This phenomenon occurred due to incomplete polymerization reaction and restrained chain propagation at short water bath time, resulting in low *η* of the polymer. As the time in the water bath increased after 4 h, the *η* of the polymer increased slightly. However, when the degree of chemical reaction was high, the prolonged reaction time did not have considerable effect on the *η* of the polymer. Therefore, the optimal water bath time was 4 h.

#### 2.1.8. Effect of Reaction Temperature on η

**Figure 8:**
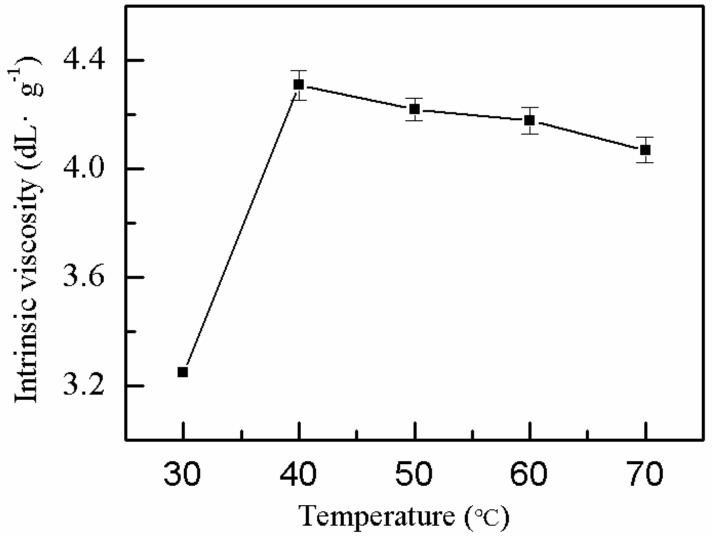
Effect of reaction temperature on *η*.

The reaction temperature is an important factor for polymerization. Therefore, the effect of reaction temperature on the *η* of P(AM–DMDAAC) was explored, whereas the other factors were constant. The *η* of the polymer exhibited a plateau with increasing reaction system temperature, as shown in Figure 8. When the water bath temperature was 40 °C, the *η* of polymer reached the maximum (4.3 dL·g^−1^) because the initiator and the activity of the monomer increased with increased reaction system temperature. The activity and the number of monomer-free radicals in the reaction system increased accordingly, and this condition was conducive to the polymerization reaction. When the temperature was very high, the polymerization reaction became increasingly intense, causing the free radicals of the monomer to crosslink and unfavorable to the polymerization reaction. Therefore, the optimal water bath temperature was 40 °C.

#### 2.1.9. Effect of pH on η

**Figure 9:**
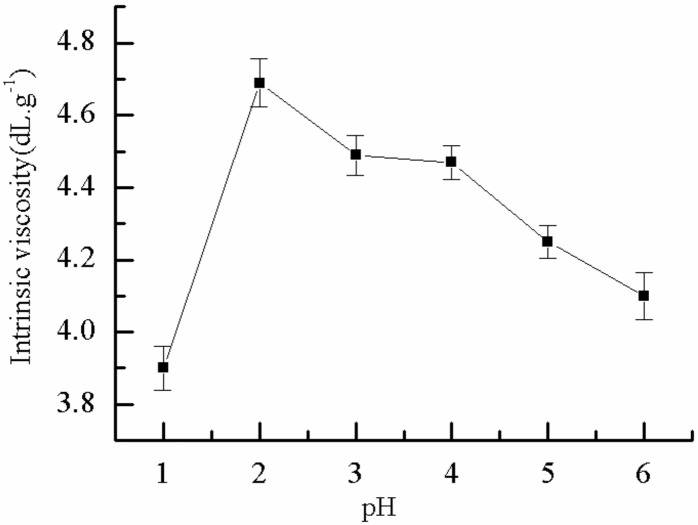
Effect of pH value on *η*.

Figure 9 displays the effect of pH on the *η* of P(AM–DMDAAC), and the other factors were constant. As shown in the image, the *η* of the polymer decreased with increasing pH. When the pH value was 2.0, the *η* of the polymer reached the maximum of 4.7 dL·g^−1^ because pH remarkably affected the initiator ammonium persulfate to counteract the speed during polymerization. When the reaction system is peracid at the pH 1.0, the amide group in monomer am will undergo an amidation reaction between or within molecules, thus cross-linking occurs between polymers, resulting in low *η* of the polymer. When pH was increased from 2.0 to 6.0, the half-life period of ammonium persulfate became shorter with higher initiation rate and temperature. The reaction heat accumulated in the reaction system, molecular chains were broken, and the *η* decreased. Therefore, the optimal pH was 2.0.

### 2.2. Characterization

#### 2.2.1. SEM of P(AM–DMDAAC)

**Figure 10:**
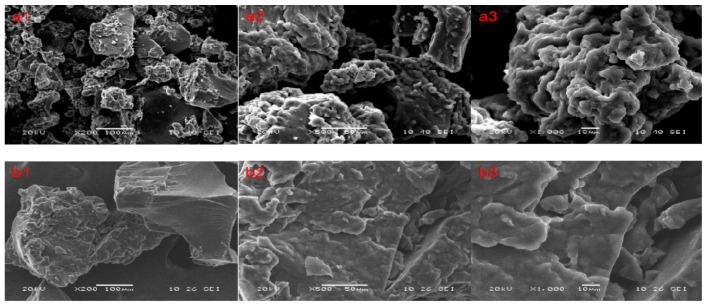
(**a**) SEM pictures of P(AM–DMDAAC); (**b**) SEM pictures of PAM.

Figure 10 illustrates the SEM images of P(AM–DMDAAC) and commercial PAM. Two different surface morphologies were observed. The structure of P(AM–DMDAAC) was very rough with a large mushroom-shaped cross-cutting structure and a comparatively irregular, uneven surface area compared with commercial PAM, which possessed a smooth and regular surface area. The difference in surface morphology between P(AM–DMDAAC) and PAM may be due to the introduction of cationic monomers and the use of MV-assisted polymerization, which induced surface modification [45]. The mushroom-shaped cross-cutting structure was conducive to enhance the ability of adsorption and bridging of P(AM–DMDAAC). Hence, numerous colloidal particles and organic pollutants in wastewater can be adsorbed.

#### 2.2.2. Infrared Spectrum of P(AM–DMDAAC)

**Figure 11:**
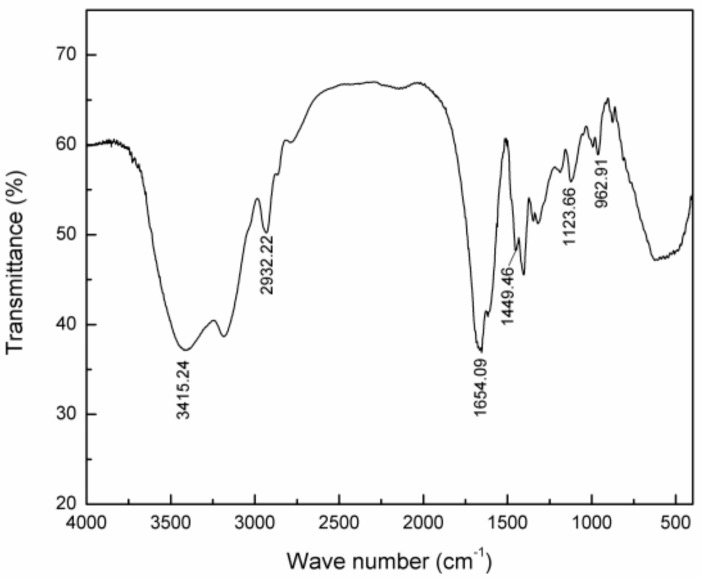
Infrared spectrum of P(AM–DMDAAC).

In Figure 11, the absorption peaks at 3415.24 and 1654.09 cm^−1^ were assigned to the stretching vibration of –NH_2_ and C=O in the AM monomer, respectively [27,46]. The peak at 2932.22 cm^−1^ represented the asymmetric absorption peak of –CH_3_ in the DMDAAC monomer, and the peak at 1449.46 cm^−1^ represented the stretching vibration absorption peak of –CH_2_– in the –CH_2_–N^+^ group of DMDAAC [47]. The peak at 962.91 cm^−1^ represented the absorption peak of –N^+^(CH_3_)_2_^−^ [48], confirming that the synthetic polymer was a copolymer of AM and DMDAAC.

#### 2.2.3. DTA–TGA of P(AM–DMDAAC)

**Figure 12:**
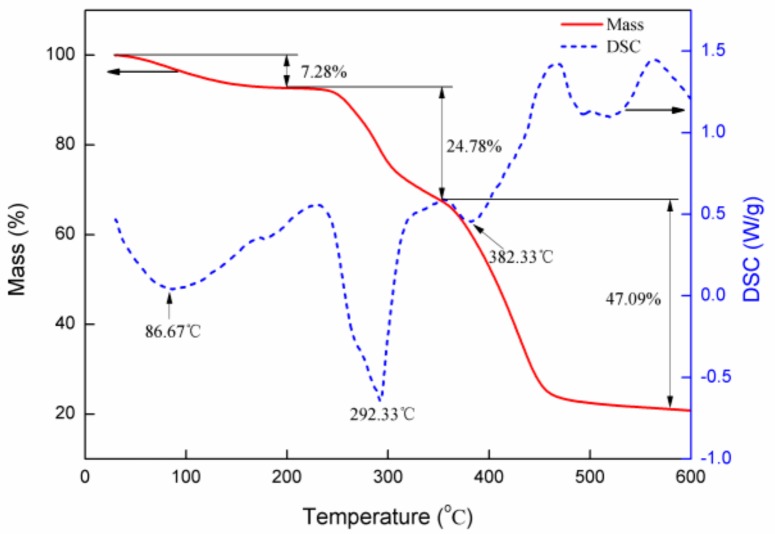
DSC-TGA of P(AM–DMDAAC).

The thermal stability of the P(AM–DMDAAC) polymer was evaluated using the DTG-60H synchronal thermal analyzer. The thermal gravimetric curve of P(AM–DMDAAC) is shown in Figure 12. With the increase in temperature, two endothermic peaks appeared in the DSC curve. The first endothermic peak observed at 30–200 °C was due to the evaporation of P(AM–DMD), and the second endothermic peak observed at 200–350 °C was due to the P(AM–DMDAAC) oxidation decomposition [49]. The TGA curves showed that with the increase in temperature, the mass changed at three points, namely, 30–200 °C with weight loss of approximately 7.28% due to the evaporation of P(AM–DMDAAC), 200–350 °C with weight loss of approximately 24.78% due to the amide–imide reaction and quaternary ammonium base on the methyl group and hydrogen chloride loss, and 350–600 °C with weight loss of approximately 47.09% due to the main chain rupture and full decomposition of the polymer [50,51]. Therefore, the DTA and TGA curves showed that the MV-assisted preparation of P(AM–DMDAAC) in normal temperature conditions was extremely stable, and decomposition would not occur under normal operating conditions.

### 2.3. Validation of the Effectiveness of P(AM–DMDAAC) in DMP Removal

#### 2.3.1. Effect of DMP Tnitial Concentration

**Figure 13:**
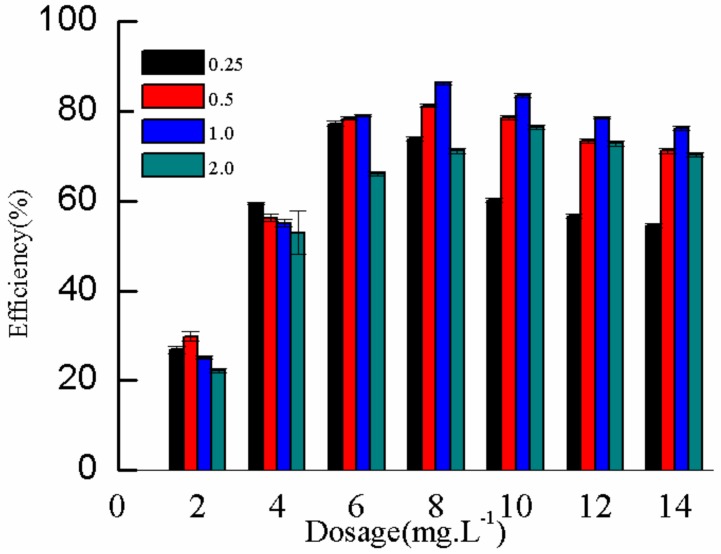
Effect of DMP initial concentration on removal rate.

In different natural waters, the concentration of DMP varies with the degree of pollution. At different initial concentrations of DMP in water, the flocculation performance will also differ. The effect of flocculant dosage on the removal rate of DMP with different initial concentrations was investigated to determine the initial concentration of DMP in simulated water. Results are shown in Figure 13. Figure 13 shows that, at an initial DMP concentration of 1.0 mg·L^−1^, the removal rate of DMP was highest (86.3%) when the dosage of the flocculant was 8 mg·L^−1^. This condition was due to the low DMP concentration in the solution. Moreover, the coagulant and the DMP molecules had less contact opportunities and low collision probability, resulting in low removal rate. With the increase in the DMP concentration in the solution, the flocculant and the DMP molecules had chances of colliding with each other, easily forming large flocs through adsorption, electric neutralization, and bridging. The formed flocs can also capture the remaining dispersed DMP molecules and smaller flocs during sinking to achieve a higher removal rate. When the concentration of DMP was further increased to 2.0 mg·L^−1^, the removal rate decreased, and the maximum removal rate was 76.5% when the dosage of flocculant was 10 mg·L^−1^. This condition was due to the high charge stability in the high-concentration solution. In an actual polluted water, the concentration of DMP was relatively low. The initial concentration of DMP was set to 1.0 mg·L^−1^ in the experiment to increase the removal rate and simulate the actual situation of pollution.

#### 2.3.2. Effect of *η*

**Figure 14:**
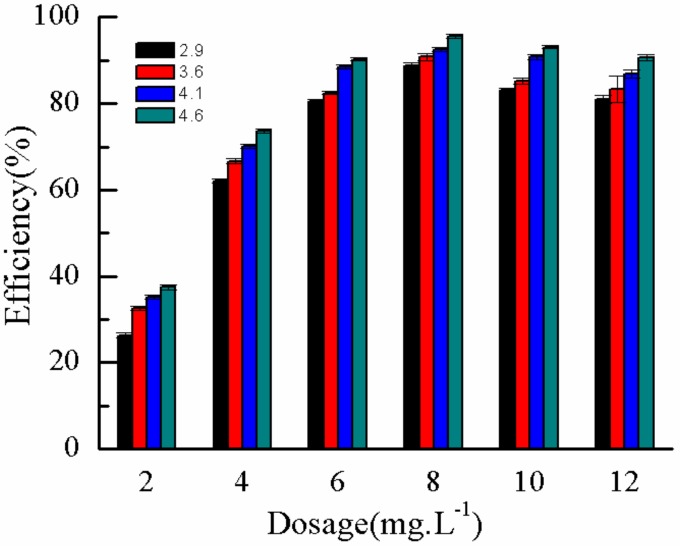
Effect of *η* on removal rate.

During flocculation, the *η* of flocculant is related to the length of molecular chain and the number of adsorption sites, affecting the removal of pollutants. In the experiment, a series of P(AM–DMDAAC) with different *η* values (2.9, 3.6, 4.1, and 4.6 dL·g^−1^) was selected as flocculants to keep other factors unchanged. The effect of different *η* flocculants on DMP removal rate was investigated under different dosages. Results are shown in Figure 14. Figure 14 shows that upon increasing the *η* from 2.9 dL·g^−1^ to 4.6 dL·g^−1^ at the same dosage, the removal rate of DMP increased, resulting in the highest removal rate of 95.6%. The possible reasons for this result were as follows. When the *η* was high, a longer molecular chain of P(AM–DMDAAC) flocculant resulted in longer molecular chain that can be adsorbed on multiple DMP molecules at the same time. Thus, more DMP molecules were easily connected by bridging and flocculation, improving the removal rate of DMP. In addition, under the same *η*, when the dosage of flocculant was low, the removal rate of DMP was low because the additional flocculant was very small to combine DMP molecules through adsorption bridging and neutralization. With the increase in dosage, the removal rate will also increase. However, to a certain extent, under high dosage, the removal rate of DMP decreased because the molecular chain of the macromolecular flocculant P(AM–DMDAAC) was tightly wrapped on the DMP molecule, resulting in a protective effect on the pollutants, which cannot be removed by adsorption bridging and neutralization. Therefore, when the concentration of DMP was 1.0 mg·L^−1^, the *η,* optimal dosage of P(AM–DMDAAC), and maximum removal rate were 4.6 mL·g^−1^, 8.0 mg·L^−1^, and 95.6%, respectively.

#### 2.3.3. Effect of Stirring Speed

**Figure 15:**
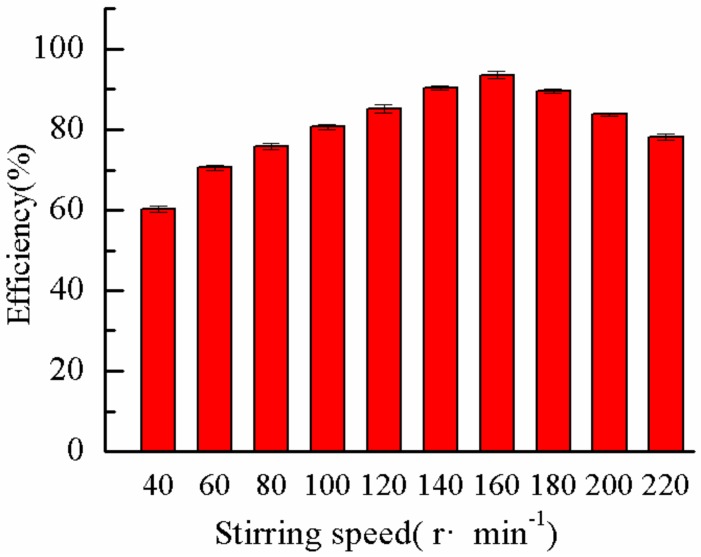
Effect of stirring speed on removal rate.

In the flocculation experiment, the mixing device adopted three different mixing procedures. The first, second, and third stages were stirred at 300 r·min^−1^ for 1 min, 160 r·min^−1^ for 4 min, and 40 r·min^−1^ for 5 min, respectively. The first stage was a high-speed stirring stage, which dispersed the flocculant into the solution quickly, increased the collision with the pollutant particles, and fully mixed them. The second stage was a medium speed stirring process, which involved the main formation and gradual growth process of flocs, and the third stage was the settling of flocs. In these three processes, the second stage was the key process, and its time was related to the whole flocculation effect. In the experiment, the stirring time was unchanged, the stirring speed of the second stage was changed, and the influence of the stirring time of the second stage on the DMP removal rate was studied. Figure 15 shows the trend of DMP removal rate at different stirring speeds. The removal rate of DMP was low when the stirring speed was low because at low stirring speed, the collision probability of DMP colloidal particles and flocculant molecules were low, and the chance of mutual combination was small, leading to the slow formation of flocs and poor settling performance. However, when the stirring speed was higher than 160 r·min^−1^, the formed floc may be broken, reducing the floc particle size. This phenomenon resulted in poor settling performance and reduced DMP removal rate. Therefore, a proper stirring speed should be ensured for the DMP colloidal particles to fully collide and combine with flocculant molecules. Very high or low values lead to the reduction of DMP removal rate. In combination with the above analysis, 160 r·min^−1^ was selected as the mixing speed in the medium-speed mixing stage.

#### 2.3.4. Effect of Stirring Time

**Figure 16:**
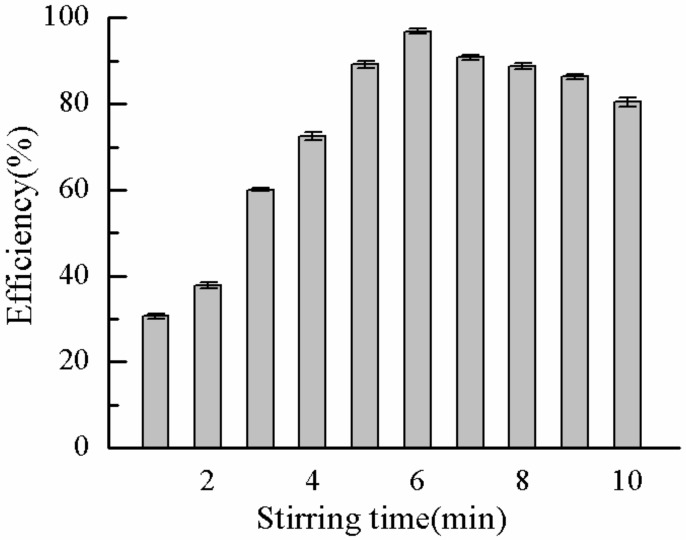
Effect of stirring time on removal rate.

In the experiment, the stirring speed of the second stage was fixed, the stirring time of the second stage was changed, and the influence of the stirring time of the second stage on the DMP removal rate was studied. The experimental results are shown in Figure 16. Figure 16 shows the trend of DMP removal rate under different stirring times of second stage. With the increase in speed stirring time, the removal efficiency of DMP also increased rapidly. In this process, the flocculant adsorbed many DMP molecules and formed a bridge. During the stirring process, the formed and dispersed small particles bridging the flocs gradually formed large flocs with other flocs, improving the settling performance and the DMP removal rate. When the stirring time reached 6 min, the removal rate of DMP reached 96.9%. At prolonged mixing time, the formed flocs are broken and dispersed into small floc particles, reducing the settling performance. After the flocs are broken, regrowth occurs. However, the particle size of the flocs after regrowth is much smaller than that before breaking, especially for the flocs formed by polymer flocculation, in which the particle size of the flocs decreases greatly. Therefore, prolonged stirring time eventually leads to the decrease in DMP removal rate. Based on the above analysis, the medium speed stirring time was set to 6 min.

#### 2.3.5. Effect of Settling Time

**Figure 17:**
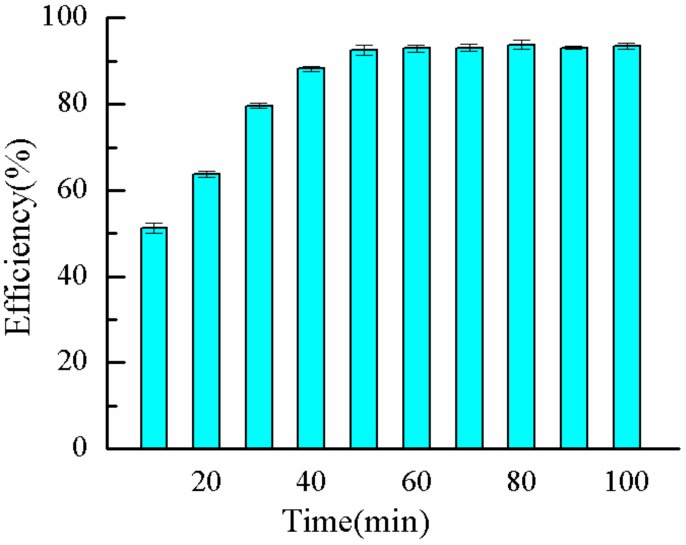
Effect of settling time on removal rate.

After coagulation and mixing, the water sample should be allowed to stand for a certain time to make the floc settle completely, and the supernatant should be obtained to detect the DMP concentration. Other factors were fixed, and the influence of settling time on DMP removal rate was investigated. Results are shown in Figure 17. Figure 17 shows that the removal rate of DMP increased rapidly within 0–30 min at the initial stage of settling. Within 30–50 min, the removal rate of DMP increased slowly, reached the maximum level, and gradually remained stable after 50 min. The main reasons for these changes were as follows. At the initial stage of sedimentation, especially at 10 min, the removal rate of DMP reached 51.3% because in the third stage of agitation, large flocs settled at the bottom of the beaker by gravity. Then, with the increase in standing time from 0 min to 30 min, the flocs that formed but did not settle in the water sample declined continuously and rapidly, and the removal rate of DMP reached 79.6%. In the process of descending, through the combination of net catching, DMP molecules remaining in the water, and the rolling and sweeping action, the flocs combined with flocs to produce larger flocs. Thus, large flocs can settle rapidly by gravity, and the DMP removal rate can be significantly improved. However, in the middle stage, DMP removal rate increased slowly due to the existence of some small flocs, small weight, and slow settling speed. After 50 min, the floc basically settled, and the DMP removal rate reached 92.6%. At the same time, the removal rate of DMP did not change much when DMP continued to settle at 100 min, indicating that the floc was stable and did not lose stability in a short time. Therefore, 50 min was the best settling time.

#### 2.3.6. Comparison of Different Flocculants

**Figure 18:**
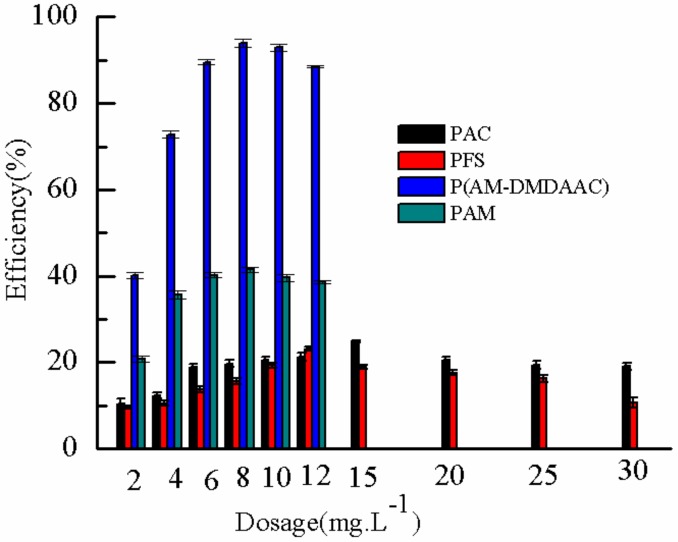
Effect of different flocculants on removal rate.

The flocculants of P(AM–DMDAAC), polyacrylamide (PAM), polyferric sulfate (PFS), and polyaluminium chloride (PAC) were used to remove DMP from the water solution and study the effect of different flocculants on DMP removal rate. Results are shown in Figure 18. Figure 18 shows that the DMP removal efficiency increased first and then decreased slightly with increasing flocculant dosage. At the same flocculant dosage, the removal rate of DMP by P(AM–DMDAAC) was highest, followed by PAM, PAC, and PFS. The removal effect of the organic flocculant was better than that of the inorganic flocculant, and the dosage was relatively low. The removal rate of DMP can reach 93.9% when the dosage of P(AM–DMDAAC) was 8 mg·L^−1^. P(AM–DMDAAC) was synthesized with AM and DMDAAC, and the monomers of DMDAAC were positively charged. Hence, the flocculant P(AM–DMDAAC) was a cationic polyacrylamide. The DMP molecule was negatively charged due to the strong electron absorption of carbonyl oxygen in the formula. Hence the P(AM–DMDAAC) can play a good role in neutralization, adsorption bridging, and net capture sweeping. The flocculant of PAM has no charge. Thus, it can only be used to remove DMP molecules through adsorption bridging and net capture sweeping. However, considering that the molecular weight of inorganic flocculant was much lower than that of the organic flocculant, the ability of adsorption bridging and net sweeping of inorganic flocculant was poor. Hence, the removal effect of DMP was relatively low.

There are some comparison of treatment effects of different coagulants on phthalate esters in water. Zheng [52] studied the flocculation performance of Anionic polyacrylamide (APAM) in the treatment of (Di-2-ethylhexyl phthalate) DEP, it was found that the removal efficiency could achieve 83.97% when the dosage of APAM was 11.01 mg/L with the initial concentration was 0.5 mg/L. Zhang [53] used Polydimethylallylammonium chloride (PDMDAAC) and Cationic polyacrylamide (CPAM) as flocculant for removing DMP in water, when PDMDAAC was used alon when the initial concnetration of DMP was 0.5 mg/L, the removal efficiency could achieve 50.29% at the dosage of PDMDAAC was 50 mg/L. When PDMDAAC and CPAM were used together with the initial concnetration of DMP was 0.5 mg/L, the removal efficiency could achieve 99.87% at the dosage of PDMDAAC was 50 mg/L, the Coagulant aid CPAM dosage was 2.5 mg/L.

## 3. Materials and Methods

### 3.1. Synthesis of P(AM–DMDAAC)

P(AM–DMDAAC) was prepared using AM and DMDAAC as reactive monomers in an aqueous solution. The reaction was carried out under MV irradiation, and the specific procedure was as follows: First, predetermined quantities of AM and DMDAAC were added into a reaction vessel. Then, deionized water was added to reach a monomer mass ratio of 30%. The mixture was stirred using a glass rod until the monomers were dissolved completely. Next, the additives (EDTA, sodium benzoate, and urea) were added to the vessel. The aqueous solution was purged with pure N_2_ at room temperature for 30 min to remove oxygen and adjust the pH value, and the MV initiator sodium benzoate was added to the vessel. The reaction vessel was introduced into the WMX-III-A Microwave reactor (Shaoguan KELI Experiment instrument Co., Ltd., Shaoguan, China) at 20 W for 1.5 min, transferred into a water bath, and dried in a vacuum oven at 80 °C. The residual concentration of DMP was determined by high performance liquid chromatography (HPLC) (LC-10AT, Shimadzu Corporation, Kyoto, Japan).

### 3.2. Characterization of P(AM–DMDAAC)

The η of the polymer, expressed in deciliters per gram (dL·g^−1^), was measured in a 1.0 mol·L^−1^ NaCl solution by using the Ubbelohde capillary viscometer (Shanghai Shenyi Glass Instrument Co. Ltd., China) at 30 ± 0.05 °C. The value of η was determined using a one-point method according to the Solomon−Ciuta formula. FTIR spectra were recorded using an FTIR spectrophotometer (Nicolet 6700, Thermo Fisher Scientific, Waltham, MA, USA) with KBr pellets, and polymer morphology was examined using SEM (JSM-6380LV, JEOL Company, Tokyo, Japan). TGA was conducted at a heating rate of 10 °C·min^−1^ under nitrogen flow of 20 mL·min^−1^ from room temperature up to 600 °C on the DTG-60H synchronal thermal analyzer (Shimadzu Corporation, Kyoto, Japan) to investigate the thermal stability of the copolymer.

### 3.3. Flocculation Test

The ZR 4-6 stirring machine (Shenzhen Zhongran Water Industry Technology Development Co., Ltd., Shenzhen, China) with six stirrers was used in this experiment. Measure 1.0 mL of DMP with a pipette, placed it in a 1000 mL volumetric flask, dissolve DMP with chromatographically pure methanol, then make up to the mark with ultrapure water, and configure a single standard stock solution which the concentration was 1000 mg/L, stored in a refrigerator at 4 °C. Dilute with ultrapure water to the required concentration when using.

An aliquot of simulated water (200 mL) was transferred into a beaker. Flocculants were dosed under medium stirring speed of 300 r·min^−1^ for 1 min and then changed to the speed of 160 r·min^−1^ for 4 min and to a final speed of 40 r·min^−1^ for 5 min. After settling for 50 min, DMP was collected below the surface for high-performance liquid chromatographic analysis.

## 4. Conclusions

A polymeric P(AM–DMDAAC) was synthesized by AM and DMDAAC through MV-assisted radiation. Several parameters affecting the performance were investigated. FTIR revealed the structural characteristics of the P(AM–DMDAAC), indicating that the synthesis of polymer by MV-assisted radiation was effective. SEM images provided the apparent morphology of the polymer, indicating its good adsorption and bridging ability compared with the commercial PAM. DTA and TGA results showed that under normal temperature condition, P(AM–DMDAAC) was extremely stable. Moreover, the reaction rate was fast, and the solubility of the P(AM–DMDAAC) was good. In the DMP removal experiments, results showed that P(AM–DMDAAC) had better flocculation than PAM, PAC, and PFS. The optimal removal rate of 96.9% can be reached at P(AM–DMDAAC) dosage of 8 mg·L^−1^ with *η* of 4.9 dL·g^−1^, second-stage stirring speed of 160 r·min^−1^, stirring time of 6 min, and settling time of 50 min.

## References

[1] Xiang Y., Gao M.-L., Ding F., Shen T. (2019). The effiffifficient removal of dimethyl phthalate by three organo-vermiculites with imidazolium-based gemini surfactants in aqueous media. Colloid Surf. A.

[2] Jing W.-W., Li D.-Q., Li J., Li X.-F., Wu Z.-H., Liu Y.-L. (2017). Photodegradation of dimethyl phthalate (DMP) by UV–TiO2 in aqueous solution: Operational parameters and kinetic analysis. Int. J. Environ. Sci. Technol..

[3] Jia H.-Z., Cao Y., Qu G.-Z., Wang T.-C., Guo X.-T., Xia T.-J. (2018). Dimethyl phthalate contaminated soil remediation by dielectric barrier discharge: Performance and residual toxicity. Chem. Eng. J..

[4] Gu J.-L., Zhao H., Liu L., Yang D., Chen H., Sun T. (2019). Investigation of the binding interactions between dimethyl phthalate and its metabolite with bovine serum albumin by multispectroscopy. Spectrochim. Acta A.

[5] Liu J., Wang K., Jia R.-B., Wang Z.-S. (2003). Removal of Esters from Drinking Water Using Ozone GAC Process. Environ. Sci..

[6] Gao X., Li H.-M., Guo J.-S., Yu Z.-X., Wang F.-T., Lu L. (2009). Removal of Phthalate Easters from Drinking Water with Zeolite Filter Column. J. Civil. Arch. Environ. Eng..

[7] Wang T.-C., Jia H.-Z., Guo X.-T., Xia T.-J., Qu G.-Z., Sun Q.-H., Yin X.-Q. (2018). Evaluation of the potential of dimethyl phthalate degradation in aqueous using sodium percarbonate activated by discharge plasma. Chem. Eng. J..

[8] Busca G., Berardinelli S., Resini C., Arrighi L. (2008). Technologies for the removal of phenol from fluid streams: A short review of recent developments. J. Hazard. Mater..

[9] Iurascu B., Siminiceanu I., Vione D., Vicente M.-A., Gil A. (2009). Phenol degradation in water through a heterogeneous photo-fenton process catalyzed by fe-treated laponite. Water Res..

[10] Zazo J.-A., Casas J.-A., Mohedano A.-F. (2009). Rodriguez JJ. Semicontinuous fenton oxidation of phenol in aqueous solution. a kinetic study. Water Res..

[11] Ispas C.-R., Ravalli M.-T., Steere A., Andreescu S. (2010). Multifunctional biomagnetic capsules for easy removal of phenol and bisphenol a. Water Res..

[12] Grabowska E., Reszczyńska J., Zaleska A. (2012). Mechanism of phenol photodegradation in the presence of pure and modified-TiO_2_: A review. Water Res..

[13] Almeida F.-T., Ferreira B.-C., Moreira A.-L., Freitas R.-P., Gil L.-F., Gurgel L.-V. (2015). Application of a new bifunctionalized chitosan derivative with zwitterionic characteristics for the adsorption of Cu(2+), Co(2+), Ni(2+), and oxyanions of Cr(6+) from aqueous solutions: Kinetic and equilibrium aspects. J. Colloid Interface Sci..

[14] Zewail T.-M., Yousef N.-S. (2014). Chromium ions (Cr6+, & Cr3+) removal from synthetic wastewater by electrocoagulation using vertical expanded fe anode. J. Electroanal. Chem..

[15] Ye S., Zhang M., Yang H., Wang H., Xiao S., Liu Y., Wang J.-H. (2014). Biosorption of Cu^2+^, Pb^2+^, and Cr^6+^, by a novel exopolysaccharide from arthrobacter, ps-5. Carbohyd. Polym..

[16] Teh C.-Y., Budiman P.-M., Shak K.-P.-Y., Wu T.-Y. (2016). Recent advancement of coagulation-flocculation and its application in wastewater treatment. Ind. Eng. Chem. Res..

[17] Kadooka H., Jami M.-S., Tanaka T., Iwata M.-J. (2016). Mechanism of clarification of colloidal suspension using composite dry powdered flocculant. J. Water Process Eng..

[18] Zhu H., Zhang Y., Yang X., Liu H., Shao L., Zhang X., Yao J. (2015). One-step green synthesis of non-hazardous dicarboxyl cellulose flocculant and its flocculation activity evaluation. J. Hazard. Mater..

[19] Zhu G.-C., Bian Y.-N., Hursthouse A.-S., Xu S.-N., Xiong N.-N., Wan P. (2020). The role of magnetic MOFs nanoparticles in enhanced iron coagulation of aquatic dissolved organic matter. Chemosphere.

[20] Sun Y.-J., Zhou S.-B., Pan S.-Y., Zhu S.-C., Yu Y., Zheng H.-L. (2020). Performance Evaluation and Optimization of Flocculation Process for Removing Heavy Metal. Chem. Eng. J..

[21] Zhu G.-C., Wang Q., Yin J., Li Z.-W., Zhang P., Ren B.-Z., Wan P. (2016). Toward a better understanding of coagulation for dissolved organic nitrogen using polymeric zinc-iron-phosphate coagulant. Water Res..

[22] Zhu G.-C., Liu J.-F., Bian Y.-N. (2018). Evaluation of cationic polyacrylamide-based hybrid coagulation for the removal of dissolved organic nitrogen. Environ. Sci. Pollut. Res..

[23] Fosso-Kankeu E., Mittal H., Waanders F., Ntwampe I.-O., Ray S.-S. (2016). Preparation and characterization of gum karaya hydrogel nanocomposite flocculant for metal ions removal from mine effluents. Int. J. Environ. Sci. Technol..

[24] Wang T., Yang W.-L., Hong Y., Hou Y.-L. (2016). Magnetic nanoparticles grafted with amino-riched dendrimer as magnetic flocculant for efficient harvesting of oleaginous microalgae. Chem. Eng. J..

[25] Weng B., Xu F., Garza G., Alcoutlabi M., Salinas A., Lozano K. (2015). The production of carbon nanotube reinforced poly(vinyl) butyral nanofibers by the forcespinning method. Polym. Eng. Sci..

[26] Craciun G., Ighigeanu D., Manaila E., Stelescu M.-D. (2015). Synthesis and Characterization of Poly(Acrylamide-Co-Acrylic Acid) Flocculant Obtained by Electron Beam Irradiation. Int. J. Pharm..

[27] Zheng H.-L., Sun Y.-J., Zhu C.-J., Guo J.-S., Zhao C., Liao Y., Guan Q.-Q. (2013). Uv-initiated polymerization of hydrophobically associating cationic flocculants: Synthesis, characterization, and dewatering properties. Chem. Eng. J..

[28] Shak K.-P.-Y., Wu T.-Y. (2017). Synthesis and characterization of a plant-based seed gum via etherification for effective treatment of high-strength agro-industrial wastewater. Chem. Eng. J..

[29] Zheng H.-L., Sun Y.-J., Guo J.-S., Li F., Fan W., Liao Y., Guan Q.-Q. (2014). Characterization and evaluation of dewatering properties of padb, a highly efficient cationic flocculant. Ind. Eng. Chem. Res..

[30] Zhang P., Ren B.-Z., Zhou Y., Liu K.-J. (2013). Removal of humic acid from aqueous solution by dimethyl diallyl ammonium chloride and acrylamide. Asian J. Chem..

[31] Li X., Zheng H.-L., Gao B.-Y., Sun Y.-J., Tang X.-M., Xu B.-C. (2017). Optimized preparation of micro-block CPAM by response surface methodology and evaluation of dewatering performance. RSC Adv..

[32] Nie R.C., Guo L.-Y., Xu C.-Y. (2008). Study on synthesis and flocculation property of cation-polyacrylamide. Int. J. Coal Sci. Technol..

[33] Sun Y.-J., Zhu C.-Y., Sun W.-Q., Xu Y.-H., Xiao X., Zheng H.-L. (2017). Plasma-initiated polymerization of chitosan-based CS-g-P(AM-DMDAAC) flocculant for the enhanced flocculation of low-algal-turbidity water. Carbohyd. Polym..

[34] Liu Z.-M., Wei Y.-L. (2011). Synthesis and Flocculating Properties of Cationic Polyacrylamide by Aqueous Two-phase Polymerization. Chin. J. Appl. Chem..

[35] Feng L., Zheng H.-L., Tang X.-M., Zheng X.-Y., Liu S., Sun Q., Wang M.-X. (2018). The investigation of the specific behavior of a cationic block structure and its excellent flocculation performance in high-turbidity water treatment. RSC Adv..

[36] Liu Y.Z., Zheng H.-L., Wang Y.-L., Zheng X.-Y., Wang M.-X., Ren J., Zhao C.-L. (2018). Synthesis of a cationic polyacrylamide by a photocatalytic surface-initiated method and evaluation of its flocculation and dewatering performance: Nano-TiO 2 as a photo initiator. RSC Adv..

[37] Xu K., Wang H.-W., Liang X.-C., Tan Y., Yao X.-P., Wang P.-X. (2018). A Novel Hyperbranched Polymeric Flocculant for Waste-Water Treatment. J. Polym. Environ..

[38] Pal S., Sen G., Ghosh S., Singh R.-P. (2012). High performance polymeric flocculants based on modified polysaccharides—Microwave assisted synthesis. Carbohyd. Polym..

[39] Zheng H.-L., Ma J.-Y., Zhu C.-J., Zhang Z., Liu L.-W., Sun Y.-J., Tang X.-M. (2014). Synthesis of anion polyacrylamide under uv initiation and its application in removing dioctyl phthalate from water through flocculation process. Sep. Purif. Technol..

[40] Hu X.-M., Mei Z.-Q., Liu H.-Y., Huang Z.-T., Wang L.-F. (1999). Study on the microwave induced synthesis of tetraphenyl porphyrin. J. South China Univ. Technol. (Nat. Sci.).

[41] Hu X.-M., Mei Z.-Q., Huang Z.-T., Lai S.-L. (1999). The synthesis of copper phthalocyanine and its sulfonat ion under microwave irradiat ion. J. South China Univ. Technol. (Nat. Sci.).

[42] Ren E.-H., Xiao H.-G., Guo R.-H., Lin S.-J. (2019). Effects of cationic degree on decolorization properties of hydrophobic cationic flocculant. Textile Dye. Finish. J..

[43] Zheng H.-L., Tang X., Shen L.-X., Gao X., Wang W., You Y.-F. (2010). Synthesis, characterization and application of cationic P(AM-DMC)used for sludge dewatering. J. Chongqing Univ..

[44] Zhang Z.-Q. (2012). Ultrasonic Assisted Synthesis of PDA and Its Characterization and Application. Master’s Thesis.

[45] Li X., Zheng H.-L., Gao B.-Y., Zhao C., Sun Y.-J. (2017). UV-initiated polymerization of acid and alkali-resistant cationic flocculant P(AM-MAPTAC): Synthesis, characterization, and application in sludge dewatering. Sep. Purif. Technol..

[46] Chen H.-X., Tang H.-M., Wu X.-Y., Liu Y.-G., Bai J.-H., Zhao F. (2016). Synthesis, Characterization, and Property Evaluation of a Hydrophobically Modified Polyacrylamide as Enhanced Oil Recovery Chemica. J. Dispersion Sci. Technol..

[47] Zhang P., Liao L.-N., Zhu G.-C. (2019). Performance of PATC-PDMDAAC Composite Coagulants in Low-Temperature and Low-Turbidity Water Treatment. Materials.

[48] Ma J.-Y., Shi J., Ding H.-C., Zhu G.-C., Fu K., Fu X. (2017). Synthesis of cationic poly-acrylamide by low-pressure UV initiation for turbidity water flocculation. Chem. Eng. J..

[49] Feng L., Liu S., Zheng H.-L., Liang J.-J., Sun Y.-J., Zhang S.-X., Chen X. (2018). Using ultrasonic (US)-initiated template copolymerization for preparation of an enhanced cationic polyacrylamide (CPAM) and its application in sludge dewatering. Ultrason. Sonochem..

[50] Ma J.-Y., Fu K., Shi J., Sun Y.-J., Zhang X.-X., Ding L. (2016). Ultraviolet-assisted synthesis of polyacrylamide-grafted chitosan nanoparticles and flocculation performance. Carbohy. Polym..

[51] Ou C.-Y., Zhang C.-H., Li S.-D., Yang L., Dong J.-J., Mo X.-L., Zeng M.-T. (2010). Thermal degradation kinetics of chitosan-cobalt complex as studied by thermogravimetric analysis. Carbohy. Polym..

[52] Zheng H.-L., Ma J.-Y., Zhai J., Zhu C.J., Tang X.-M., Liao Y., Sun Y.-J. (2014). Optimization of flocculation process by response surface methodology for diethyl phthalate removal using anionic polyacrylamide. Desalin. Water Treat..

[53] Zhang P., Zheng H.-L., Deng X.-L., Jiang S.-J., Wang J.-J., Liu L., Yang B. (2011). RemoVal of Dmethyl Phthalate from Water with Enhanced Coagulation Technology. J. Civil Arch. Environ. Eng..

